# Chemosensitization of U-87 MG Glioblastoma Cells by Neobavaisoflavone towards Doxorubicin and Etoposide

**DOI:** 10.3390/ijms23105621

**Published:** 2022-05-17

**Authors:** Mateusz Maszczyk, Klaudia Banach, Marta Karkoszka, Zuzanna Rzepka, Jakub Rok, Artur Beberok, Dorota Wrześniok

**Affiliations:** Department of Pharmaceutical Chemistry, Faculty of Pharmaceutical Sciences in Sosnowiec, Medical University of Silesia in Katowice, Jagiellońska 4, 41-200 Sosnowiec, Poland; d200888@365.sum.edu.pl (M.M.); kbanach@sum.edu.pl (K.B.); d200971@365.sum.edu.pl (M.K.); zrzepka@sum.edu.pl (Z.R.); jrok@sum.edu.pl (J.R.); abeberok@sum.edu.pl (A.B.)

**Keywords:** antineoplastic agents, doxorubicin, etoposide, glioblastoma, isoflavones

## Abstract

Glioblastoma (GB) is the most common type of glioma, which is distinguished by high mortality. Due to the rapid progression of the tumor and drug resistance, the treatment is often ineffective. The development of novel therapies in a big part concerns the application of anti-cancer agents already used in clinical practice, unfortunately often with limited effects. This could be overcome through the use of compounds that possess chemosensitizing properties. In our previous work, it has been shown that neobavaisoflavone (NBIF) enhances the in vitro activity of doxorubicin in GB cells. The aim of this study was a further investigation of the possible chemosensitizing effects of this isoflavone. The experimental panel involving image cytometry techniques, such as count assay, examination of mitochondrial membrane potential, Annexin V assay, and cell cycle analysis, was performed in human glioblastoma U-87 MG cells and normal human astrocytes (NHA) treated with NBIF, doxorubicin, etoposide, and their mixes with NBIF. NBIF in co-treatment with etoposide or doxorubicin caused an increase in the population of apoptotic cells and prompted alterations in the cell cycle. NBIF enhances the pro-apoptotic activity of etoposide and doxorubicin in U-87 MG cells, which could be a sign of the chemosensitizing properties of the isoflavone.

## 1. Introduction

Gliomas account for 75% of malignant primary neoplasms of the central nervous system (CNS), and most of these cases are glioblastoma (GB) [1]. The incidence rate of this tumor is highest in the Caucasian population (5.1 cases per 100,000 people) and increases with age [2]. The paramount hallmark of GB is high mortality—the 5-year survival rate is lower than 10%, and a median remaining lifetime of diagnosed patients spans from 0.5 to 1.5 years [3]. The typical treatment is based on a resection of the tumor with a safe surgical margin, followed by radiotherapy and chemotherapy with temozolomide [1,4]. Unfortunately, mainly due to drug resistance and rapid progression of GB, this regimen is ineffective in most patients, and the tumor inevitably recurs [2,5].

Over the past decades, researchers made efforts to develop a novel therapy for GB. The research is focused, among others, on immunotherapy [6,7]. Although this kind of treatment seems to be promising for many types of neoplasms, such as melanoma or non-small cell lung cancer, clinical trials to date have shown that immunotherapy does not improve prognosis in patients with GB [6]. This is due to the local immune dysfunction and the immunosuppressive properties of the tumor [7]. Until now, the only monoclonal antibody approved for use in GB treatment is bevacizumab, an antibody directed against vascular endothelial growth factor (VEGF). However, it does not affect the overall survival in patients [8]. Another approach to new therapeutic strategies in GB is the application of already known anti-cancer agents, such as topoisomerase inhibitors (e.g., doxorubicin or etoposide) [9]. Their efficacy has been proved in pre-clinical and clinical studies, mainly in combined treatment with other chemotherapeutics [10,11,12,13]. However, there are major obstacles that challenge the use of these chemotherapeutics in the treatment of GB, i.a., poor permeability of the blood-brain barrier [14]. Advances in drug delivery methods offer a promise to improve the bioavailability of topoisomerase inhibitors in the brain [14,15,16]. Another hurdle is the induction of drug resistance [17]. This can be overcome by sensitization of the tumor to the anti-cancer agents [18]. A combined therapy involving a chemical that targets one of the mechanisms of chemoresistance, including drug uptake, its metabolism, or cell death and survival, is expected to improve pharmacotherapy efficacy. The compounds that possess such properties are polyphenols [19].

Polyphenols are a large group of natural substances including flavonoids, lignans, stilbenes, phenolic acids, and other polyphenols [20,21]. They are known for their numerous beneficial properties, i.a., anti-oxidative, anti-inflammatory, and anti-diabetic effects [21]. They also exert anti-cancer activity, which includes anti-proliferative, anti-metastatic, and pro-apoptotic actions in tumor cells. This is due to the fact that polyphenols directly affect signaling pathways essential for cell growth and survival, such as mitogen-activated protein kinase (MAPK), phosphatidylinositol-3-kinase (PI3K)/protein kinase B (AKT), and mammalian target of rapamycin (mTOR) pathways [22]. In several studies, it has been shown that the combined regimen of a chemotherapeutic and a polyphenol causes an increase in the treatment efficiency [23,24,25,26]. For example, curcumin through restraint of PI3K/AKT and nuclear factor kappa B (NF-κB) pathways and inhibition of apoptosis suppression makes cancer cells sensitive to several anti-cancer agents, i.e., fluorouracil and oxaliplatin [25]. Similar properties are also found with a soy isoflavone genistein, which sensitizes ovarian [24] and cervical [26] cancer cells towards cisplatin, and breast cancer cells towards tamoxifen [23] in the in vitro models. Resveratrol is another polyphenol that exerts chemosensitizing properties—it has been shown that this compound sensitizes multiple cancer cell lines, i.a., lung carcinoma, leukemia, prostate cancer, and pancreatic cancer to various anti-neoplastic agents, such as paclitaxel, cisplatin, doxorubicin, and cytarabine [27].

According to the literature, doxorubicin or etoposide in combination with polyphenols exert greater anti-tumor effects in the in vitro models [28,29,30,31,32,33]. In our previous study [34], we demonstrated that neobavaisoflavone (NBIF), a *Psoralea corylifolia* L. polyphenol, has a moderate effect on viability and growth in human U-87 MG glioblastoma cells. Simultaneously, we have also discovered that it increases the impact of doxorubicin on cell viability when combined with it. These findings lead us to investigate other features connected with the possible chemosensitizing properties of NBIF.

The aim of this study was to examine the effect of NBIF in combination with etoposide or doxorubicin on cell growth, apoptosis, and the cell cycle in human U-87 MG glioblastoma cells. For comparative purposes, identical treatments were performed in normal human astrocytes (NHA).

## 2. Results

### 2.1. NBIF in Combined Treatment with Doxorubicin Causes a Reduction in Number of U-87 MG Cells

**Figure 1:**
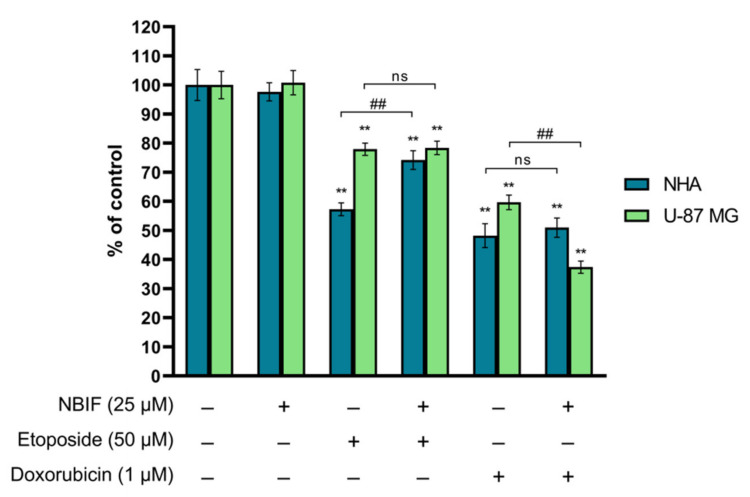
The effect of NBIF alone and in combination with etoposide and doxorubicin on the growth of NHA and U-87 MG cells. Cell cultures were treated with NBIF (25 μM), etoposide (50 μM), doxorubicin (1 μM) and in mixes: etoposide + NBIF (50 μM + 25 μM) and doxorubicin + NBIF (1 μM + 25 μM) for 48 h. The results are presented as percent of corresponding control. ** *p* < 0.001 vs. control; ## *p* < 0.001 for comparisons between groups; ns—not significant.

In order to investigate the effect of NBIF on the activity of etoposide and doxorubicin in NHA and U-87 MG cells, the assessment of cell number was performed. In the presented experiments, the conditions (concentrations of drugs and the isoflavone, and the time of their exposure to cell cultures) that had been used in our previous study [34] were applied. The number of cells was estimated using the NucleoCounter^®^ NC-3000™ fluorescence image cytometer, as described in Section 4.4. The results have shown that NBIF (25 μM) alone does not affect the number of NHA or U-87 MG cells (Figure 1). There was also no difference between U-87 MG cells treated with etoposide (50 μM) alone or in combination with NBIF—the cell growth in these groups was estimated at approx. 78% as compared to the control. However, it weakened the action of the chemotherapeutic on the growth of astrocytes by ca. 17%. This effect was not found in NHA cells treated with doxorubicin or doxorubicin + NBIF mix. In U-87 MG, the combined treatment of doxorubicin and NBIF resulted in a decrease in cell growth to approx. 37% of the control. In comparison to the drug alone, this reduction was by ca. 22%.

### 2.2. Co-Treatment of NBIF with Etoposide or Doxorubicin Intensifies Apoptosis in U-87 MG Cells and Astrocytes

**Figure 2:**
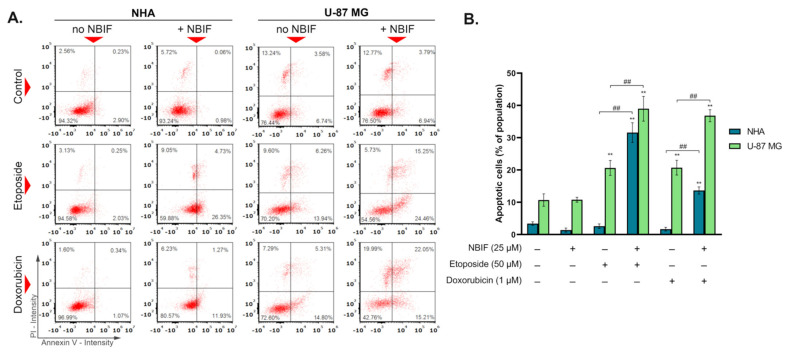
The results of Annexin V assay after 48 h of the incubation of NHA human astrocytes and U-87 MG glioblastoma cells with etoposide (50 μM) or doxorubicin (1 μM) alone and in combination with NBIF (25 μM). (**A**) Representative scatter plots present cell population divided into four quadrants: healthy cells (lower left), early apoptotic cells (lower right), late apoptotic cells (upper right) and non-apoptotic dead cells (upper left). (**B**) Mean values and SD of percentage of the annexin V-positive cells (bar graph) from three independent experiments in at least triplicate. The results are presented as percent of whole population. ** *p* < 0.001 vs. control; ## *p* < 0.001 for comparisons between groups. PI—propidium iodide.

The Annexin V assay allows detection of phosphatidylserine translocation to the outer surface of the cell, indicating apoptosis. To explore the effect of NBIF (25 μM) on the apoptosis induction by etoposide (50 μM) and doxorubicin (1 μM), NHA (normal human astrocytes) and U-87 MG human glioblastoma cells were incubated for 48 h with chemotherapeutics alone and in co-treatment with NBIF. The obtained results (Figure 2) revealed that the addition of the isoflavone causes a significant increase in the apoptotic subpopulation in all groups treated with a drug + NBIF mix. The strongest effect was noticed in U-87 MG cells treated with etoposide + NBIF (ca. 39% of the whole population) and doxorubicin + NBIF (ca. 37% of the whole population). Nevertheless, an intense effect was also found in NHA cells treated with etoposide combined with NBIF–the subpopulation of apoptotic cells in this group was estimated at approx. 32%. The lesser impact was observed in astrocytes treated with doxorubicin + NBIF mix, where it was approximately 14%.

### 2.3. The Impact of NBIF Combined with Etoposide or Doxorubicin on the Mitochondrial Membrane Potential in NHA and U-87 MG Cells

**Figure 3:**
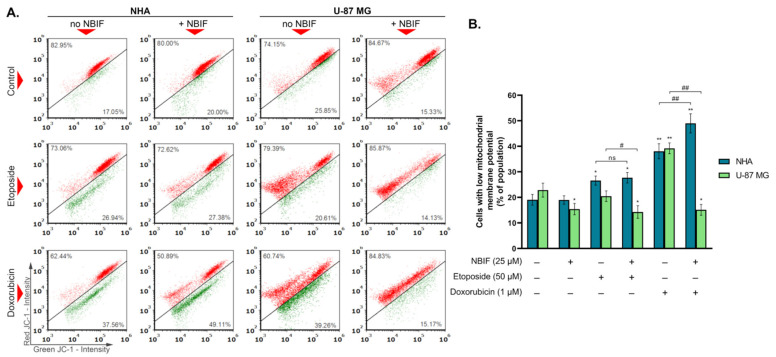
The mitochondrial membrane potential of NHA and U-87 MG cells treated for 48 h with etoposide (50 μM) or doxorubicin (1 μM) and their mixes with NBIF (25 μM). (**A**) Representative scatter plots present cells divided into two subpopulations: cells with polarized mitochondrial membrane (red), and cells with depolarized mitochondrial membrane (green). (**B**) Mean values and SD of percentage of cell subpopulation characterized by low mitochondrial membrane potential (bar graph) from three independent experiments in at least triplicate The results are presented as percent of whole population. * *p* < 0.05, ** *p* < 0.001 vs. control; # *p* < 0.05, ## *p* < 0.001 for comparisons between groups; ns—not significant.

Mitochondrial membrane potential disruption is often related to the early stage of apoptosis [35]. To detect these changes in cells treated with etoposide (50 μM) or doxorubicin (1 μM) and in their combinations with NBIF (25 μM), staining with fluorescent dye JC-1 was performed. In polarized (healthy) mitochondria, JC-1 accumulates in its polymeric form exhibiting red fluorescence, whereas, in depolarized mitochondria, it localizes in the cytoplasm in a monomeric form characterized by green fluorescence. Therefore, a decrease in the red-to-green fluorescence intensity ratio indicates depolymerization of the mitochondrial membrane. The results (Figure 3) showed that there is no significant difference between NHA cells treated with etoposide and etoposide + NBIF mix (approx. 27% and 28%, respectively). In contrast, the doxorubicin + NBIF mix caused an 11% increase in the subpopulation of astrocytes with depolarized mitochondria (to ca. 49%), in comparison to doxorubicin alone (ca. 38%). An opposite effect was found in U-87 MG, where the percentage of cells with disrupted mitochondrial potential was lower in groups treated with drug + NBIF mixes than with only a chemotherapeutic and was at the similar level as in U-87 MG cells treated with NBIF alone (approximately 15%).

### 2.4. Etoposide- or Doxorubicin + NBIF Co-Treatment Prompts Cell Cycle Alterations in U-87 MG Cells

**Figure 4:**
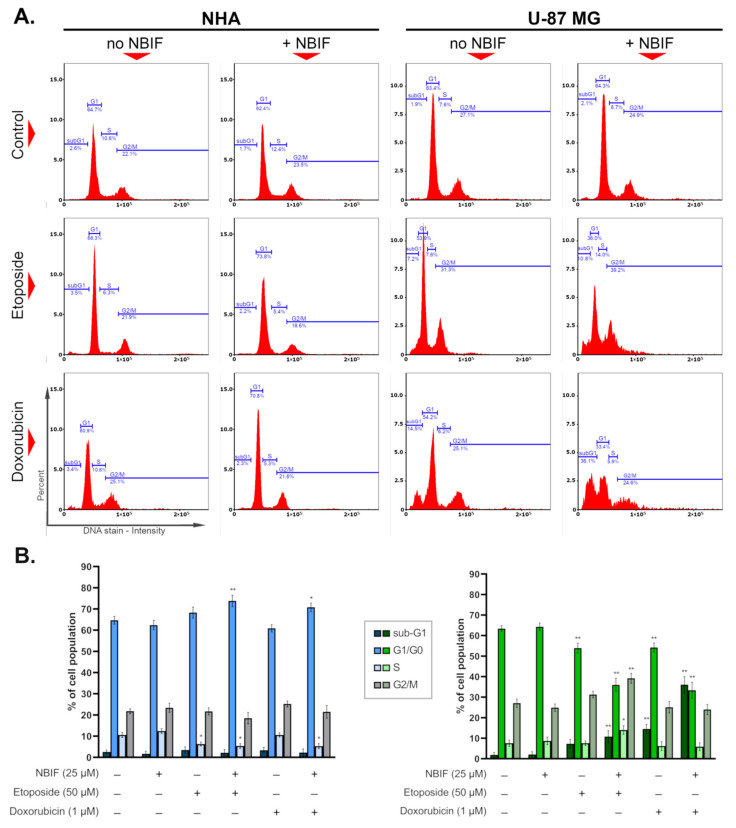
The influence of etoposide (50 μM), doxorubicin (1 μM) and their mixes with NBIF (25 μM) on the cell cycle profile in NHA and U-87 MG cells (48 h of treatment). (**A**) Representative histograms showing the distribution of cells with different DNA content in the cell cycle phases. (**B**) Mean values and SD of percentage of cell subpopulations in different cell cycle phases (bar graphs: left–NHA cells, right–U-87 MG cells) from three independent experiments in at least triplicate The results are presented as percent of corresponding control. * *p* < 0.05, ** *p* < 0.001 vs. control.

The impact of etoposide (50 μM), doxorubicin (1 μM), and their mixes with NBIF (25 μM) on the cell cycle in NHA astrocytes and U-87 MG glioblastoma cells was investigated after 48 h of treatment. The obtained results (Figure 4) showed a slight effect on the astrocyte cell cycle, where significant changes were found in the G1/G0 phase of cells incubated with etoposide + NBIF and doxorubicin + NBIF combinations—these regimens caused an increase by ca. 9% and 6% relative to the control, respectively. In contrasting, co-treatments led to notable alterations in the cell cycle of U-87 MG cells. A common feature of these groups was a decrease in the number of cells in the G1/G0 phase, by approx. 30% of the control in the population treated with doxorubicin + NBIF mix, and 28% of the control in the U-87 MG cells incubated with etoposide + NBIF. In the latter group, an elevation in S and G2/M phases was also noticed, which was by ca. 6% and 12%, sequentially. In turn, another thing that was found in U-87 MG cells solely was an increase in the percentage of sub-G1 cells treated with doxorubicin alone, in its combination with NBIF, and etoposide + NBIF mix. These were by approximately 13%, 34%, and 9% relative to the control, respectively.

## 3. Discussion

Among all gliomas, GB is the most common type of tumor [1,2]. Despite aggressive, multi-faceted treatment, it is very often fatal as the GB relapses in most patients [2,3,5]. Due to ineffective therapy, there is an urgent need to develop a novel regimen that would be able to overcome such obstacles as GB tumor heterogeneity or drug resistance. One of the therapeutical strategies that are being taken into consideration is a combined treatment targeting multiple pathways crucial for neoplastic cell survival [18,19]. This is the basis of the process called chemosensitization, which assumes the use of a non-toxic compound that is able to enhance the activity of a chemotherapeutic [27,36]. The vast majority of identified chemicals that exhibit such properties are phenolic substances, such as polyphenols [36]. In this study, the sensitizing effect of NBIF in the GB in vitro model towards doxorubicin and etoposide was explored.

The anti-cancer effects of NBIF have been demonstrated in several different tumor cell lines [34,37,38,39,40], including gliomas [34,38]. Previously, we have found that this isoflavone moderately decreased the viability of U-87 MG cells in a concentration-dependent manner, and slightly reduced the percentage of living cells at 100 μM [34]. We hypothesized that NBIF may affect cell proliferation in a cell cycle-independent manner, as it did not cause cell death. In our last study, we also performed the cell viability assay in U-87 MG cells treated in combined treatments of NBIF (25 μM, 100 μM) and a topoisomerase inhibitor: irinotecan (10 μM), etoposide (50 μM) or doxorubicin (1 μM), to find if the isoflavone affects the activity of these drugs. We discovered that only the cells treated with doxorubicin + NBIF mixture had lower viability than the group incubated with the drug alone. The current study was aimed at further exploration of the possible chemosensitizing effect of NBIF at the cellular level. We conducted an experimental panel consisting of cytometric analysis of U-87 MG cells incubated with NBIF-drug mixes, with all used topoisomerase inhibitors, in the same conditions as used previously (concentrations and exposition time). However, during the research, irinotecan + NBIF treatment was excluded since no effects were observed when compared to a chemotherapeutic alone.

The programmed cell death known as apoptosis is a complex process involving multiple signaling pathways, which are important for the proper functioning of the organism [41]. It can be activated by such stimuli as DNA damage, hypoxia, oxidative stress, or immune-related factors [41,42]. The typical changes to a cell that occur during apoptosis are, i.a., phosphatidylserine externalization and DNA fragmentation [41]. The alterations in apoptosis often result in cancerogenesis, as the deregulation of this process leads to uncontrollable cell growth and proliferation, and further tumor progression [41]. For this reason, induction of apoptosis is the main target of modern cancer pharmacotherapy [42]. The anti-cancer drugs that are utilized as chemotherapeutics affect various points of the cellular machinery intending to trigger cell death. A big group of them blocks DNA replication, targeting specific elements like topoisomerases [43]. In our study, etoposide (50 μM) and doxorubicin (1 μM) caused phosphatidylserine translocation in approx. 20% of U-87 MG cells, while in astrocytes no effect was observed (Figure 2B). In groups treated with drug + NBIF mixture, there was an almost 2-fold increase in the percentage of apoptotic subpopulations (early and late apoptotic) of U-87 MG cells. Moreover, a combination of a drug with NBIF caused an elevation in the sub-G1 phase subpopulation (Figure 4B), which are late apoptotic cells with fragmented DNA [42]. The largest change was observed in the doxorubicin + NBIF group (ca. 2.6-fold, when compared to doxorubicin alone). An increase in apoptotic subpopulations between groups treated with the drug alone and its mix with NBIF was also found in normal cells (Figure 2B). Despite the large difference in the Annexin V assay, the percentages of subpopulations with fragmented DNA were not significantly different from the control (Figure 4B), which means that there was no late apoptosis in drug + NBIF-treated astrocytes.

Mitochondria have a central role in the activation and regulation of the intrinsic pathway of apoptosis. When the programmed cell death is initiated, cytochrome C and other pro-apoptotic factors are released into the cytoplasm. Once there, cytochrome C forms with other proteins a complex called apoptosome. This leads to the activation of the initiator caspase-9, which starts the apoptotic signaling cascade. The process of cytochrome C release is accompanied by changes in mitochondrial membrane potential, which becomes disrupted [35]. To investigate whether NBIF enhances apoptosis in U-87 MG cells in combined treatments through mitochondria, we performed the analysis of their membrane potential (Figure 3). We found that in every group of GB cells where NBIF was present, the subpopulation of cells with decreased mitochondrial membrane potential was lower than in the control (Figure 3B). However, this was not connected with the cell death as there were more PI-positive cells in the drug + NBIF mix-treated U-87 MG cells (Figure 2A). This could mean that NBIF enhances apoptosis independently from mitochondria. The studies of Szliszka et al. [37] and Kim et al. [38] proved that this isoflavone is able to enhance cell death triggered by tumor necrosis factor-related inducing ligand (TRAIL), which is unrelated to the mitochondria apoptotic pathway. Alternatively, the decrease in the subpopulation with low mitochondrial membrane potential in U-87 MG cells treated with NBIF could mean that this isoflavone prevents mitochondrial membrane disruption in this cell line, beside the apoptosis intensification. It may be connected, i.a., with mitochondrial respiratory chain, mitochondrial DNA or mitochondria-related apoptotic factors [44]. In turn, we also had shown that NBIF did not affect the mitochondria membrane potential in astrocytes, except in the cells treated with a doxorubicin + NBIF mix. However, it had a limited effect on apoptosis induction (Figure 2B). This is consistent with the fact that normal cells are less prone to mitochondrial damage than tumor cells [44]. On the other hand, the observed effect of NBIF on mitochondria in U-87 MG cells might have contributed to the results that we received previously from the WST-1 test, which is based on the activity of mitochondrial dehydrogenases [34]. This could be a reason for no differences between the groups treated with etoposide and its mix with the isoflavone.

The cell cycle comprises mitosis (M) and interphase, which can be further subdivided into G1, S, and G2 phases. When a cell exits its replicative cycle, it enters the resting state, a G0 phase. Each stage of the division cycle is characterized by the specific processes that occur within a cell—in the G1 phase, the cell grows and prepares for the DNA synthesis, which takes place in the S phase. In the G2 phase, it continues to grow and prepares for the M phase, where the cell division happens [45]. Tumor cells are usually characterized by the dysregulated cell cycle, which results in intensified divisions and abnormal proliferation [45,46]. The interruption of its progression is frequently associated with the activation of apoptosis [46]. Several anti-cancer agents exhibit phase-specific activity against cells, causing an arrest. Topoisomerase inhibitors, like etoposide or doxorubicin, are considered to block the succession of the S or G2/M phase [47]. In the presented study, at used concentrations, etoposide and doxorubicin did not cause this effect in U-87 MG cells (Figure 4B). Instead, the reduction in G1/G0 phases was found, which in the doxorubicin-treated cells was in favor of the sub-G1 subpopulation (late apoptotic cells). This effect was exacerbated in doxorubicin + NBIF, which also caused an inhibition of cell growth (Figure 1). In turn, in GB cells incubated with etoposide + NBIF, the decrease in G1/G0 phase was in favor of the S and G2/M phases (Figure 4B), which could mean that the isoflavone enhances the phase-specific activity of etoposide. The minor changes in astrocyte cell cycle distribution were found in etoposide-, etoposide + NBIF- and doxorubicin + NBIF-treated cells. In the second-mentioned group, NBIF slightly limited cell growth inhibition (Figure 1). This might be evidence of the small chemoprotective properties of NBIF.

In several studies, it has been proven that polyphenols can modulate the effects of doxorubicin and etoposide in neoplastic cells [28,29,30,31,32,33]. In general, they were found to intensify drug-induced apoptosis and cell cycle arrest. In the research conducted by Mahbub et al. on leukemia cells, it was shown that the combination of doxorubicin or etoposide with apigenin, emodin, cis-stilbene, or quercitin enhances apoptosis through the activation of caspase-3, and intensifies the S and/or G2/M phase arrest [30]. Similar results were retrieved in studies conducted on human breast cancer cells [28,32,33], which demonstrated the sensitization towards doxorubicin by resveratrol. In turn, rhamnetin, a flavonol, was shown to increase the cell cycle alterations in hepatocellular carcinoma caused by etoposide [31]. Compounds structurally analogous to NBIF and classified as isoflavones were also demonstrated to exhibit anti-neoplastic drug activity-modulating properties. For instance, Xue and colleagues [29] demonstrated that genistein, a soy isoflavone, influences doxorubicin activity, increasing the apoptotic subpopulation and the cell cycle arrest in the G2/M phase in human breast cancer cells. 

Although our study is limited to one tumor cell line, we believe that it gives a strong basis for further research. This is supported by the fact that the present study was conducted on the normal counterpart of GB cells. Our future investigations regarding possible chemosensitizing properties of NBIF will concern a different glioma cell line. We will also aim at exploring the underlying molecular mechanism.

## 4. Materials and Methods

### 4.1. Chemicals and Reagents

Neobavaisoflavone (7-hydroxy-3-(4-hydroxy-3-(3-methyl-2-buten-1-yl)phenyl)-4H- -1-benzopyran-4-one), dimethyl sulfoxide (DMSO) and penicillin G were retrieved from Sigma-Aldrich Inc. (St. Louis, MO, USA). Gibco Astrocyte Medium (DMEM, N-2 Supplement, One Shot fetal bovine serum), Trypsin/EDTA solution were purchased from ThermoFisher Scientific (Waltham, MA, USA). Dulbecco’s modified Eagle’s medium (DMEM) and fetal bovine serum (FBS) were obtained from Cytogen (Zgierz, Poland). Via1-Cassettes™, NC-Slides™ A2 and A8, as well as Solution 3 (1 μg/mL DAPI, 0.1% Triton X-100 in PBS), Solution 7 (200 μg/mL JC-1), Solution 8 (1 μg/mL DAPI in PBS), Solution 15 (500 μg/mL Hoechst 33342), Solution 16 (500 μg/mL propidium iodide) were acquired from ChemoMetec (Lillerød, Denmark). Annexin V binding buffer and Annexin V-CF488A conjugate were purchased from Biotium (Fremont, CA, USA). Neomycin sulfate was retrieved from Amara (Kraków, Poland). In the study, the following drugs were used: etoposide (Etoposid-Ebewe, EbewePharma, Ahmedabad, India), and doxorubicin (Doxorubicin Accord, Accord, Ahmedabad, India). The rest of the chemicals were purchased from POCH S.A. (Gliwice, Poland).

### 4.2. Cell Cultures

Gibco Human Astrocytes (NHA), acquired from ThermoFisher Scientific (Waltham, MA, USA), were cultured in Gibco Astrocyte Medium consisting of DMEM, N-2 Supplement and One Shot fetal bovine serum. Human glioblastoma cells (U-87 MG), obtained from Sigma-Aldrich Inc. (St. Louis, MO, USA), were maintained in DMEM medium with the addition of FBS. All growth media were supplemented with amphotericin B (0.25 mg/mL) neomycin (10 μg/mL), and penicillin G (10,000 U/mL). Cell cultures were incubated in humidified 5% CO_2_ atmosphere at 37 °C.

### 4.3. Cell Treatment

Before the treatment, cells were seeded in T-75 flasks (NHA: 0.3 × 10^6^ cells/flask; U-87 MG: 1 × 10^6^ cells/flask). The incubation with neobavaisoflavone (NBIF; 25 μM), doxorubicin (1 μM), doxorubicin + NBIF (1 μM + 25 μM), etoposide (50 μM), etoposide + NBIF (50 μM + 25 μM) lasting 48 h was started 48 h and 24 h after inoculation for NHA and U-87 MG cells, respectively. Subsequently, cells were trypsinized, centrifuged and resuspended in the proper medium in order to conduct further analyses.

### 4.4. Cell Count Assay

To determine the total number of cells, image cytometry technique was used. After the treatment with tested substances, non-fixed cells were detached by trypsinization and loaded into Via1-Cassette™ (ChemoMetec), which contains the fluorescent dyes: DAPI (4′,6′-diamidino-2-phenylindole) staining dead cells and acridine orange (AO) staining whole cell population. Samples were immediately analyzed with NucleoCounter^®^ NC-3000™ fluorescence image cytometer (ChemoMetec) controlled by NucleoView NC-3000 Software (ChemoMetec).

### 4.5. Annexin V Assay

In order to detect apoptotic cells, the Annexin V assay was conducted. The method is based on the ability of fluorescent Annexin V conjugate to bind to phosphatidylserine, a lipid that is translocated to the outer surface of the plasma membrane during apoptosis. After the treatment and trypsinization, a total of 0.4 × 10^6^ cells in each sample were suspended in 100 μL of Annexin V binding buffer, then 2 μL of Solution 15 (500 μg/mL Hoechst 33342, staining whole population) and 2 μL of Annexin V-CF488A conjugate were added. Following the incubation (15 min at 37 °C), stained cells were centrifuged (400× *g* for 5 min) and washed twice with Annexin V binding buffer. Eventually, the cell pellets were resuspended in 100 μL of Annexin V binding buffer supplemented with 2 μL of Solution 16 (500 μg/mL propidium iodide, staining dead cells). Samples were loaded into NC-Slides™ A2 and analyzed with NucleoCounter^®^ NC-3000™ fluorescence image cytometer.

### 4.6. Mitochondrial Potential Assay

The mitochondrial membrane potential was evaluated via JC-1 (5,5′,6,6′-tetrachloro-1,1′,3,3′-tetraehtylbenzimidaziolocarbocyanine iodide) staining and measured using image cytometry technique. To the cell suspensions (1 × 10^6^ cells/mL), Solution 7 (200 μg/mL JC-1) was added and incubated for 15 min at 37 °C. After washing with PBS, cells were resuspended in Solution 8 (1 μg/mL DAPI in PBS) and analyzed immediately with NucleoCounter^®^ NC-3000™ fluorescence image cytometer.

### 4.7. Cell Cycle Assay

The distribution of cell cycle phases was estimated using NucleoCounter^®^ NC-3000™ fluorescence image cytometer, by the DNA content measurement. After the treatment and trypsinization, cells were resuspended in PBS (1 × 10^6^ cells/mL) and fixed with 70% cold ethanol for at least 2 h. Subsequently, cells were stained with Solution 3 (1 μg/mL DAPI, 0.1% Triton X-100 in PBS), and after 5 min of incubation at 37 °C loaded into NC-Slides A8™ and analyzed using NucleoCounter^®^ NC-3000™ fluorescence image cytometer.

### 4.8. Statistical Analysis

All results are presented as mean values of at least three separate experiments conducted in triplicate ± SD. Differences between groups were evaluated using two-way ANOVA followed by post-hoc Tukey’s test. A significant difference was considered at *p*-value lower than 0.05. Statistical analysis of the results was performed in GraphPad Prism 8.0 (GraphPad Software, San Diego, CA, USA).

## 5. Conclusions

In the present paper, we investigated cellular features of possible chemosensitizing properties of NBIF. It has been demonstrated that in combination with one of the two toposiomerase inhibitors: doxorubicin or etoposide, the isoflavone caused an increase in the apoptotic subpopulation. Our results suggest that this was triggered in a mitochondria-independent manner. NBIF was also found to enhance drug-induced cell cycle arrest.

In parallel, the study was also conducted on normal cells, which were astrocytes. Altough NBIF prompted an increase in the drug-induced apoptosis, it showed limited effect on their proliferation and the cell cycle in comparison to drugs alone.

## Data Availability

The data that support the findings of this study are available from the corresponding author upon reasonable request.
